# Insights into Arbovirus Evolution and Adaptation from Experimental Studies

**DOI:** 10.3390/v2122594

**Published:** 2010-12-02

**Authors:** Alexander T. Ciota, Laura D. Kramer

**Affiliations:** 1 The Arbovirus Laboratories, Wadsworth Center, New York State Department of Health, Slingerlands, NY 12159, USA; E-Mail: aciota@wadsworth.org; 2 University at Albany, State University of New York, Albany, NY 12222, USA

**Keywords:** arbovirus, viral evolution, viral fitness, host cycling, mutant swarm dynamics

## Abstract

Arthropod-borne viruses (arboviruses) are maintained in nature by cycling between vertebrate hosts and haematophagous invertebrate vectors. These viruses are responsible for causing a significant public health burden throughout the world, with over 100 species having the capacity to cause human disease. Arbovirus outbreaks in previously naïve environments demonstrate the potential of these pathogens for expansion and emergence, possibly exacerbated more recently by changing climates. These recent outbreaks, together with the continued devastation caused by endemic viruses, such as Dengue virus which persists in many areas, demonstrate the need to better understand the selective pressures that shape arbovirus evolution. Specifically, a comprehensive understanding of host-virus interactions and how they shape both host-specific and virus-specific evolutionary pressures is needed to fully evaluate the factors that govern the potential for host shifts and geographic expansions. One approach to advance our understanding of the factors influencing arbovirus evolution in nature is the use of experimental studies in the laboratory. Here, we review the contributions that laboratory passage and experimental infection studies have made to the field of arbovirus adaptation and evolution, and how these studies contribute to the overall field of arbovirus evolution. In particular, this review focuses on the areas of evolutionary constraints and mutant swarm dynamics; how experimental results compare to theoretical predictions; the importance of arbovirus ecology in shaping viral swarms; and how current knowledge should guide future questions relevant to understanding arbovirus evolution.

## 1. Introduction

Arthropod-borne viruses (arboviruses) are unique in that they require cycling between disparate hosts, *i.e.*, vertebrates and hematophagous arthropod vectors. Arboviruses are predominately RNA viruses in the families *Flaviviridae, Togaviridae, Bunyaviridae, Rhabdoviridae*, and *Reoviridae*; yet a single genus in the family *Orthomyxoviridae* (*Thogotovirus*) and a single DNA virus in the family *Asfarviridae* (*African swine fever virus*) are also included among the arboviruses. The fact that these viruses are almost exclusively RNA viruses may be explained by a requirement for significant plasticity in order to succeed in dynamic host environments [1]. RNA-dependent RNA-polymerase (RdRp) error rates are estimated to range from 10^−3^ to 10^−5^ errors / nucleotide / round of replication [2,3]. This, together with rapid and high levels of viral replication, allows quick exploration of fitness landscapes and production of variants which may have an advantage in different host environments.

Arboviruses are responsible for causing a significant public health burden throughout the world, with over 100 species of virus having the capacity to cause human disease. Among these, the majority are mosquito-borne viruses including flaviviruses such as *Dengue virus* (DENV), *Yellow fever virus* (YFV), *Zika virus* (ZIKV), and the Japanese encephalitis serogroup viruses such as *West Nile virus* (WNV), *St. Louis encephalitis virus* (SLEV), and *Japanese encephalitis virus* (JEV); alphaviruses including *Eastern equine encephalitis virus* (EEEV), *Western equine encephalitis virus* (WEEV), *Venezuelan equine encephalitis virus* (VEEV), *Sindbis virus* (SINV), *Ross River virus* (RRV), and *Chikungunya virus* (CHIKV); and bunyaviruses including *Lacrosse virus* (LACV), *Rift Valley fever virus* (RVFV), and *California encephalitis virus* (CEV). Some human arboviral pathogens such as *Colorado tick fever virus* (CTFV), *Crimean-Congo hemorrhagic fever virus* (CCHV), *Louping Ill virus* (LIV), and *Tick-borne encephalitis virus* (TBEV), are primarily, if not exclusively, transmitted by ticks. Additional invertebrate vectors including biting midges and sandflies, among others, also have been implicated in transmission of arboviruses with public health significance [4]. Greater than 14,000 species of blood-sucking insects have been recognized as capable of arbovirus transmission [5]. Most human disease resulting from arboviruses is a consequence of spillover from enzootic cycles, although humans act as amplifying hosts in ‘urban’ cycles of such arboviruses as DENV, YFV, ZIKV, and CHIKV. Many of the zoonotic viruses are also highly pathogenic to their nonhuman vertebrate hosts, leading to significant disruptions in wild bird and mammal populations. Other zoonotic viruses such as *blue tongue virus* (BTV), *African horse sickness virus* (AHSV), *Vesicular stomatitis virus* (VSV), *epizootic hemorrhagic disease virus* (EHDV), and LIV generally do not cause significant human disease but do cause considerable disease in both wild and livestock populations and consequently have led to significant ecological and economic disruptions [6,7].

Recent arbovirus outbreaks have demonstrated the potential of these viruses to emerge and expand their range, many as a consequence of changing climates and landscapes [8]. The impact of climatic factors has been well noted for RVFV [9]; and DENV continues to expand its range as a result of changing landscapes [7]. One of the best documented cases of an arbovirus invading a naïve habitat and successfully establishing itself is WNV. Since its introduction to the New York City area in 1999, WNV steadily increased both its host and geographic range, spreading across the U.S. and into Canada, Mexico, and Central and South America [10–16]. Worldwide, WNV has infected over 75 species of mosquitoes [17] and over 300 species of birds [18]. In the U.S. alone, WNV has been confirmed in over 40,000 people and caused significant declines in some avian populations [19,20]. In 1996, the alphavirus *O’nyong-nyong virus* (ONNV) emerged in Uganda following a 35 year absence and caused widespread disease [21]; and in 2000, RVFV cases were documented for the first time outside of Africa [22]. A close relative of ONNV, CHIKV, emerged in Kenya in 2004 and spread to the islands of the Indian Ocean in 2005, resulting in an outbreak in which over one million human cases of chikungunya fever were reported in previously naïve populations [23]. Other arboviruses of veterinary importance, such as Usutu virus and BTV, have recently emerged for the first time in Europe and had significant effects on wildlife and livestock populations [24,25].

These recent outbreaks, together with the continued devastation caused by viruses such as DENV, YFV, and JEV, which remain endemic throughout their geographic range, demonstrate the need to better understand the selective pressures that shape arbovirus evolution and emergence. Specifically, a comprehensive understanding of host-virus interactions and the role of host-specific and virus-specific evolutionary pressures is needed to fully evaluate the factors that govern the potential for host shifts and geographic expansions. One approach to advance our understanding of the factors influencing arbovirus emergence and evolution is the use of experimental studies in the laboratory. Here we review the contributions that laboratory passage and experimental infection studies have made to the field of arbovirus adaptation and evolution. In particular, this review focuses on the areas of evolutionary constraints and mutant swarm dynamics, how experimental results compare to theoretical predictions, the importance of arbovirus ecology in shaping viral swarms, and how current knowledge should guide future questions relevant to understanding arbovirus evolution.

## 2. The Cost of Host Cycling

Despite the enormous potential for sequence change inherent in RNA viruses, the consensus sequences of most arboviruses have remained highly genetically conserved in nature [26–33]. This evolutionary stasis is generally attributed to the differential selective pressures applied by disparate vertebrate and invertebrate hosts [34,35]. This implies that only mutations which are either beneficial or neutral in both hosts become fixed, resulting in a situation in which sequence changes are much more likely to be purged by purifying selection than in single host systems [36–38]. Indeed, phylogenetic studies of arboviruses analyzing the proportion of nonsynonomous change over time demonstrate that purifying selection is generally the dominant selective force in arbovirus evolution [39,40]. An extension of the concept of genetic constraints is limitation on host-specific adaptation, *i.e.*, fitness trade-offs. The generally accepted theory is that cycling between disparate hosts selects for generalists and, as a consequence, arboviruses sacrifice the ability to be host specialists [41,42]. Specifically, arboviruses are hypothesized to lack host specialization as it would result in either positive selection for changes which are advantageous to one host but would be detrimental in the alternate host (antagonistic pleiotropy), or the accumulation of neutral mutations in one host which would be detrimental in the alternate host (mutational accumulation) [43].

Although these concepts are generally accepted, experimental studies have provided mixed results in testing the hypotheses that (a) significant constraints on genetic change of arboviruses result from host cycling, and (b) arboviruses are subject to significant fitness trade-offs as a consequence of host cycling. Here we review the contributions of such studies, beginning with *in vitro* experimental evolution studies. These studies, although lacking the complexity of natural *in vivo* systems, have been useful tools in beginning to define the selective pressures acting on arboviruses in a simpler setting.

### 2.1. Flaviviruses

The flavivirus genome is single-stranded, positive sense RNA which is approximately 11 kb in length with a single long open reading frame [44]. The genus *Flavivirus* consists of more than 70 species, but the virus which undoubtedly has had the most widespread impact on public health, with annual worldwide infections approaching 100 million, is DENV [45]. A previous study with DENV-2 demonstrated that no consensus change occurred with sequential passage in mosquito (*Aedes albopictus*, C6/36) cell culture, and only modest consensus change occurred with sequential passage in mammalian (African green monkey kidney, Vero) cell culture [46], but it should be noted that only a 2.5 kb region of the viral genome was sequenced in this study. This work also demonstrated that the mammalian cell derived viral strains generally grew to slightly higher titers in mammalian cell culture, whereas mosquito derived viral strains generally grew to slightly lower titers in mammalian cell culture. Conversely, a more recent study with DENV-2, evaluating full genome sequences and fitness changes after sequential or alternate passage in mammalian and mosquito cell lines, did not produce evidence that cycling results in host specific fitness trade-offs [47]. Both DENV-2 studies found that fewer genetic changes were seen in consensus sequences from the mosquito cell derived virus relative to the vertebrate cells or cycled strains, supporting the idea that, at least in cell culture, it is replication in invertebrate cells rather than host cycling that may dampen genetic change. Given the obvious limitations of cell culture work, it is not clear if these results can be extrapolated to natural host systems, yet evaluation of sequence variation of DENV-3 from naturally infected *Ae. aegypti* mosquitoes and humans also found generally less sequence variation in mosquito-derived isolates [48]. Although the latter study also suggested similar trends could be noted in the mutant swarm, neither of the DENV *in vitro* passage studies evaluated the mutant spectra of experimentally passed strains, a step which is crucial for a comprehensive evaluation of genetic change. In fact, similar passage studies with WNV and SLEV [49] demonstrated that limited sequence change was fixed in the consensus following 40 passages in mosquito cell culture, yet when mutant swarm diversity was evaluated for mosquito cell derived WNV, it revealed the genetic change was substantial despite a lack of consensus change [50]. Similar to the Vasilakis *et al.* 2009 DENV study, these studies demonstrated that both WNV and SLEV are capable of significant host-specific adaptation with sequential passage in mosquito cells, yet this seemed to come at little cost in the ‘bypassed’ vertebrate host. Taken together, results from *in vitro* flavivirus studies do not support the idea that limitations on fixed, consensus change result from cycling alone, nor do they generally support the existence of significant fitness trade-offs resulting from host cycling.

### 2.2. Alphaviruses

The majority of togaviruses are mosquito-borne viruses in the genus *Alphavirus* and many of the experimental evolution studies have focused on important pathogens within this genus [1]. The alphavirus genome is similar to that of the flaviviruses in that it is single stranded, positive sense RNA, approximately 11 kb in length, yet unlike flaviviruses, it has two ORFs, one full-length genomic responsible for translation of nonstructural proteins, and one truncated subgenomic which is responsible for translation of structural genes. In comparison to flavivirus studies*, in vitro* passage studies with alphaviruses provide somewhat contradictory results regarding the extent of both fitness trade-offs and evolutionary constraints. A study with EEEV in which virus was passaged sequentially in either vertebrate (BHK) or invertebrate (C6/36) cells, or in alternate hosts, reported that fitness increases were measured in cell lines used for sequential passage and fitness losses were generally seen in bypassed cells [51]. Despite this, virus which was cycled accrued fitness gains in both cell types which were equivalent to the levels reported in sequentially passed strains. Here, strains derived from sequential passage did accumulate more consensus genetic change than cycled strains, leading to the conclusion that evolutionary rates, but not necessarily host-specific fitness, were constrained by host cycling. A subsequent study with EEEV performed similar passage using a more ecologically appropriate vertebrate cell line [avian; Peking duck embryo (PDE)] [52]. Although genetic change was not evaluated in this study, phenotypic results indicated again that alternation of hosts selected for viruses well adapted for both hosts, with no substantial cost in terms of viral growth or infectivity, measured relative to the magnitude of specialization achieved through sequential passage. Despite this, these studies were also the first to clearly demonstrate unbalanced selective pressures in disparate hosts, with increased infectivity measured in insect cells but not avian cells following alternate passage. A study with SINV also demonstrated that adaptations in terms of relative fitness to both host environments were achievable through cycling [53]. In this study, fitness gains in alternately passed strains were generally less than those measured in sequentially passed strains; however, some cycled strains achieved host-specific gains equivalent to those sequentially passed. Sequentially passed strains did generally accrue a cost in the bypassed host yet this demonstrated that SINV has the ability to achieve specialization in spite of cycling. In addition, consensus genetic change was on average less in cycled strains relative to single host strains. Overall, *in vitro* passage studies with alphaviruses demonstrate that host specialization through sequential passage often result in fitness costs in the bypassed host, and that host cycling may dampen the rate of consensus genetic change; yet these studies also show that host specialization without significant fitness trade-offs is at times attainable through cycling.

### 2.3. Rhabdoviruses

VSV, a positive sense, single-stranded RNA virus with 5 distinct genes (ORFs), is the most studied arbovirus in the field of experimental evolution; ironically VSV may not be highly representative of arboviruses in general. Unlike the mosquito-borne flaviviruses and alphaviruses already discussed, which generally have a narrow vector range, a broad range of vectors and modes of transmission have been implicated for VSV. Sandflies, as well as biting midges and mosquitoes play dominant roles in VSV maintenance and transmission [54,55], and other arthropods have also been implicated including black flies [56] and grasshoppers [57]. The capacity for VSV to be transmitted mechanically by a vector as well as nonsystemically has further complicated understanding VSV epidemiology [4,58]. In essence, VSV may be the ultimate generalist capable of exploiting numerous ecological niches.

Studies by Holland and colleagues with VSV [59], and subsequent studies with foot and mouth disease virus (FMDV) [60] detailed methods for evaluating relative fitness which became the experimental standard for many arbovirus evolution studies. This work, together with contributions by Duarte *et al.* [61] and Clarke *et al.* [62], was the first to demonstrate the remarkable mutability and phenotypic plasticity of VSV using *in vitro* passage and subsequent evaluation of fitness changes. Novella *et al.* [63] demonstrated significant adaptation of VSV to sandfly cells with persistent passage in these cells in conjunction with substantial declines in both viral fitness in vertebrate cells and mouse neurovirulence. Although no genetic analyses were done, this was the first study to consider the importance of replicative strategy (persistent *vs.* acute) in shaping arbovirus adaptation. These results supported the concept of fitness trade-offs with host-specific adaptation. In work by Turner and Elena [64], fitness trade-offs with sequential passage were again demonstrated for VSV, yet similar to alphavirus studies, it was shown that host cycling could also achieve equivalent host specific fitness gains. In a subsequent study, consensus genetic change following similar sequential or alternate passage series was determined [65]. In contrast to what had been shown previously for EEEV and SINV [51,53], the results demonstrated that the number of mutations accumulated during alternate passage was similar or larger than the number accumulated during sequential passage, counter to the idea that slow rates of evolution in nature are a consequence of host cycling. This study also did not demonstrate any significant fitness trade-offs as seen with previous studies, leading to further questions of the relative importance of the cell type *versus* replicative strategy. A follow-up study investigated this concept and demonstrated that the persistent phase of the cycle (invertebrate) is the dominant evolutionary force and that trade-offs are dependent on strategy and not necessarily host cell type for VSV *in vitro* [66]. The idea that the invertebrate is the dominant force in VSV evolution was further confirmed by the sequencing of populations generated in the Turner and Elena study. The results confirmed that strains subject to alternating passage shared many more substitutions with strains passed exclusively in invertebrate cells than they did with those derived from vertebrate passage [67]. Taken together, this body of work demonstrates not only that cycling does not necessarily constrain host-specific adaptations, but also that host shifts do not necessarily constrain genetic change, at least in the case of VSV. In addition, it clearly demonstrates that vertebrate and invertebrate environments do not represent equal partners in shaping arbovirus evolution.

### 2.4. In vivo Studies

The fact that some studies, even with the same virus, yield different results points to the importance of the experimental conditions in the various *in vitro* passage studies. The appropriateness of many factors including multiplicities of infection, temperatures, number of passages, length of individual passages, measures of viral fitness, and source of the passed virus strains, is not always clear, yet slight variations in these factors may have profound effects on outcomes. Additionally, studies with both alphaviruses [68] and flaviviruses [69] demonstrate non-specific adaptation to heparin sulfate as a receptor *in vitro.* These specific examples demonstrate the general fact that *in vitro* systems are often inapt representatives of natural environments and that experimental passage studies which utilize relevant *in vivo* systems more closely mimicking natural environments are needed. In 1975, Taylor and Marshall demonstrated that RRV rapidly evolved to increased virulence when sequentially passed in mice; however, alternate passage between *Ae. aegypti* mosquitoes and mice constrained changes in virulence [70]. Since these studies, *in vivo* evolution studies have been generally lacking, yet recent work with flaviviruses WNV and SLEV [71–74] and the alphavirus VEEV [75] have again begun to test the validity of *in vitro* findings in relevant *in vivo* hosts. Sequential passage of VEEV in vertebrates (mice or hamsters) or *Ae. aegypti* mosquitoes, led to specialized viruses in each host, whereas alternating passage did not result in fitness gains in either host, supporting the idea that cycling constrains host-specific adaptation. Although in this study the presence of potentially important mutant variants was not evaluated, consensus genetic change associated with host-specific adaptations were modest and no greater in number than changes identified in virus subjected to alternate passage. While this demonstrates the ability for further host specialization, these results do not support the idea that rate of evolutionary change is constrained by host cycling. Experimental passage of WNV in *Cx. pipiens* mosquitoes revealed the capacity for WNV to adapt further to this host, yet no measurable cost was demonstrated in terms of replicative ability in chickens [72]. Similar studies with SLEV demonstrated, quite surprisingly, that further gains in replicative ability are not achievable in *Cx. pipiens* following passage by inoculation. Since release from host alteration does not lead to further adaptation in this study, it suggests, unlike VEEV and WNV work, that SLEV adaptation to mosquitoes in nature may not be significantly hampered by host cycling. These studies also demonstrate that significant adaptation to avian hosts already exists, but some gains in terms of infectivity were possible. An important caveat to WNV and SLEV studies is the use of intrathoracic inoculation rather than bloodfeeding for mosquito passage. Infection, replication, and dissemination from the mosquito midgut may require variants different from those selected for infection of and replication in parenteral tissues; yet to address this experimentally is difficult, as viral titers generally are not sufficiently high to infect a large proportion of the mosquitoes via bloodfeeding without intermediate amplification. This problem was overcome with VEEV by the pooling of mosquitoes [75], which risks providing a slightly artificial representation of true cycling. Despite the problems inherent in these *in vivo* studies, they provide a much better representation of the complexity of the selective pressures to which arboviruses in nature are subject than do *in vitro* studies. The fact that even this limited body of work provides results that are not wholly in agreement demonstrates that it may not be possible to use a broad brush to generalize the mechanisms by which arboviral hosts shape the viral population.

### 2.5. Conclusions

Although variability exists between the results of arboviral passage studies completed thus far, there are general conclusions pertaining to host adaptation, viral fitness and viral evolution that are broadly supported. In regards to both the genetic and phenotypic consequences of host cycling, studies by in large refute the inevitability of fitness trade-offs, *i.e.*, the idea that cycling should always result in suboptimal adaptation in each host. Arboviruses in the lab and in nature undoubtedly have the capacity to achieve high levels of adaptation to both host environments in spite of cycling; and host-specific adaptations often carry no cost in alternate environments. Although some constraints on host specific adaptation certainly exist, they are often subtle and are species-dependent. This is not surprising since arboviruses differ not only in host utilization but also in genome organization, rates of recombination, breadth of mutant swarms, mechanisms of transmission, and mechanisms of seasonal survival (all addressed in detail below). A complete understanding of how such factors shape arbovirus populations is crucial to understanding arbovirus evolution and epidemiology. Beyond species specific differences, one also must look deeper at gene specific differences. Studies with VSV demonstrate that changes in particular regions result in antagonistic pleiotropy in divergent hosts whereas other mutations may be neutral or co-adaptive in other hosts [67,76]. The idea that some mutations which increase viral fitness in one host are neutral in another, demonstrates that one mechanism by which trade-offs can be avoided is by the differentiation of genes that are functional in different hosts. Furthermore, the fact that some mutations can be beneficial in different environments suggests another possible mechanism by which fitness trade-offs are avoided; some genes and their products interact with their hosts in a very generic manner which make seemingly different environments indistinguishable. One example of this is the level of specificity in cell surface receptor/viral antigen binding. The VSV G protein has demonstrated the ability to initiate entry into all cell types tested to date and therefore is often exploited for gene transfer and gene therapy [77]. This property is likely directly related to the broad host range and often elusive ecology of VSV. An additional mechanism by which a virus can evade trade-offs is by exploitation of the pliability of the viral mutant swarm, whose dynamic nature is visited below.

Although it is clear that rates of genetic change in nature are generally low relative to their potential, results from experimental evolution studies as a whole do not support the hypothesis that this slow accumulation of change is a result of host cycling alone. In fact, most studies have demonstrated the same modest accumulation of fixed consensus change occurs with sequential passage and that selective pressure in individual hosts, rather than host alternation, is more likely responsible for the slow rates of evolution in nature. The main caveat to this conclusion is that the majority of these studies consider only consensus level change. Furthermore, modest change in terms of numbers of mutations is not always synonymous with the phenotypic impact of change. Single substitutions can have profound effects on replicative ability and/or infectivity in particular hosts; this has not only been observed experimentally, but also in nature. Genotypes of VEEV associated with outbreaks have been shown to have single mutations in the E2 gene responsible for increased vector competence [78] or equine virulence [79]. In the U.S. from 2001 to 2004, the NY99 genotype of WNV was fully displaced by a newly emergent genotype, WN02 [29,80]. This genotype, despite being defined by just two synonomous and one nonsynonomous change relative to NY99, was found to be transmitted earlier and more efficiently by *Culex* mosquitoes [81] and this displacement occurred in concert with the explosive expansion westward of WNV across the U.S. Similarly, the recent outbreaks of CHIKV in the islands of the Indian Ocean were associated with the emergence of new viral strains that shared a single common substitution in the E1 envelope gene in conjunction with a variable second mutation [82–84], increasing vector competence of *Ae. albopictus* mosquitoes [85–87]. These examples highlight the pliability of arboviral pathogens which, despite slower than predicted evolutionary rates, still have the capacity to readily produce variants that can be exploited in new environments.

## 3. The Role of the Arbovirus Mutant Swarm

Arboviruses often exist as a collection of variable genomes within a host. This mixed population of genomic variants, collectively referred to as the mutant swarm or mutant spectrum, is the result of a rapid replication rate combined with the error prone nature of viral RdRps. Although many refer to this swarm as a ‘quasispecies’ structure, the origin of the term quasispecies [88] describes not just a collection of genetic variants in flux but, rather, a molecular state defined by specific conditions [89]. Evaluating the quasispecies theory requires that variants exist in an equilibrium state, which is likely to be rare during viral infections due to variable selective pressures and bottlenecks, particularly for arboviruses. Nonetheless, the quasispecies theory is highly relevant to a review of the biological implications of the arbovirus mutant swarm, since it is Eigen’s ideas that brought the idea of coupled populations into the mainstream rather than individual wildtype entities. It is now generally accepted that for RNA viruses it is not a single species but, rather, an entire distribution of variants which itself will act as the unit of selection in any given environment [90–93], although some question the validity of this concept in nature [94]. The size and genetic diversity of a particular mutant swarm is governed by a dynamic balance between mutation and selection, but in order to fully understand how selection acts on these populations one must first fully describe the role of the mutant swarm both within and among hosts.

### 3.1. Adaptability

One clear advantage diverse mutant populations possess is phenotypic plasticity and adaptability to new and dynamic environments. It seems that this adaptability may indeed be required for all RNA viruses, as recent studies with poliovirus have demonstrated that high fidelity mutants that are constrained in their capacity for exploration of sequence space are often highly attenuated, and therefore, promising vaccine candidates [95–97]. Conversely, it has been shown that RNA viruses exist on the precipice of an error threshold which, if crossed, sends them into extinction [98–99]. This concept has led to exploration of lethal mutagenesis following antiviral treatment, such as with the antiviral drug ribavirin, which incorporates into the RdRp and has been shown to increase the error rate beyond the error threshold [101–104]. Although ribavirin has been demonstrated to be effective against some arboviruses [105–107], the mechanism by which this mutagen acts on these viruses may be independent of error catastrophe [108,109]. Presently it is unclear how effective lethal mutagenesis is as an antiviral strategy for arboviruses.

Phenotypic plasticity is a characteristic of highly diverse populations, which is particularly important for arboviruses that replicate in both highly divergent hosts and diverse tissues within each host. Extreme fitness losses of VSV in the vertebrate environment resulting from passage in sandfly cells can be almost completely reversed with a single passage in BHK cells, a result that plainly demonstrates the ability of the viral mutant swarm to maintain variants in a population which have proven useful in the past [63]. This ability to maintain mammalian ready variants in the VSV mutant swarm even after up to a year of persistence in sandfly cells was further confirmed in a subsequent study [106]. This concept also has been demonstrated with HIV [107,108] and FMDV [109,110] where it has been termed ‘molecular memory’, another mechanism by which arboviruses may be capable of host cycling with little indication of consensus level evolution or constraint on host-specific adaptation.

Selective pressures that arboviruses encounter in vertebrate and invertebrate systems are undoubtedly very different. In contrast to what has been shown for DENV-3 [46], intrahost genetic diversity of WNV derived from mosquitoes in nature was found to be substantially more heterogeneous than WNV derived from vertebrate hosts [115]. This host-dependent nature of mutant swarm size was confirmed with passage studies in the laboratory for both WNV and SLEV and, in the case of WNV, differences were attributed to relaxed purifying selection in mosquitoes [71,73]. A recent study demonstrated that these differing selective pressures could be attributed to differing immune pressures within each host. Specifically, the most diverse portions of the WNV genome were synonymous with the portions most likely to be targeted by RNA interference (RNAi) in *Culex* mosquitoes [116]. In a subsequent study using artificially diverse WNV strains, it was confirmed that high levels of intrahost genetic diversity were associated with increased fitness in *Cx. quinquefasciatus* mosquitoes [74]. While it is not clear if the levels of intrahost diversity found in nature are sufficient to confer a similar advantage, these studies reveal another possible mechanism by which high mutation rates are advantageous for arboviruses and demonstrate that selection for diversity, rather than diversity as simply a consequence of relaxed selection may exist in invertebrate hosts. Although vertebrate immune responses to arboviruses have been studied extensively, the field of insect immunity is still in the early stages. Recently, there have been significant advances in the understanding of invertebrate viral immunity, particularly in the area of RNAi [117]. The RNA-imediated pathway has now been implicated in modulating infection either directly or indirectly of DENV, ONNV, SINV, and WNV in invertebrate vectors [118–121]. It has also become evident in recent years that arboviral infections are often not benign to vectors and that the magnitude and scope of pathology is variable depending on the virus and invertebrate species [122,123]. A more complete understanding of the antiviral response, including both virus- and host-specific differences is crucial if we are to better describe the selective pressures that act on arboviruses in their invertebrate hosts.

### 3.2. Viral Fitness

In conjunction with the benefit of adaptability which may result from increases in mutant swarm breadth, a role for minority variants in viral fitness is also well defined [60,113,124]. Increases in VSV fitness were seen with no change identified in the consensus sequence [125]. Similarly, the importance of the mutant swarm in fitness of cell culture adapted strains of WNV also has been demonstrated [50]. Specifically, a highly significant fitness increase in mosquito cells was accompanied by just two nonsynonomous substitutions in the WNV consensus sequence and reverse genetics experiments demonstrated that consensus changes alone could not produce the adaptive phenotype. Despite this, an accumulation of a sizable mutant swarm was seen during the passage series which created these adapted strains, which stands in contrast to what one would expect to observe with positive selection of adapted variants, and thus further implicates the swarm in fitness gains. The WNV mutant swarm has also been implicated in viral pathogenesis in mice, where increases in mutant swarm breadth were associated with decreases in both mouse morbidity and mortality [71]. What remains unclear is what interactions among the variants in the mutant swarm allow a combination of minority variants to produce a dominant phenotype. Epistatic relationships within arbovirus genomes are well documented [126] but the extent to which interactive relationships among genomes exist has not been fully defined. One mechanism by which interaction occurs is by genome recombination and reassortment, yet the occurrence of these events in arboviruses, although variable among individual species, is generally low. Although WEEV appears to have resulted from a recombination event between EEEV and a SINV-like ancestor [127–129], there exists no other evidence of heterologous recombination of alphaviruses, and the frequency of homologous recombination within individual species of alphaviruses appears to be very limited [1]. For flaviviruses, homologous recombination has been reported for DENV and JEV, yet no such evidence exists for YFV [130–132]. A recent examination of all known WNV whole genome sequences did find evidence of recombination in one strain of WNV, yet the overall analysis indicated that it is unlikely that recombination significantly contributes to genetic variation of WNV [133]. In contrast, because their genomes are segmented, bunyaviruses have been found to undergo reassortment [134–136], demonstrating the importance of genome organization in producing genetic variation. These species specific differences need to be considered when evaluating the implications of mutant swarm dynamics.

Intriguing evidence exists for cooperative interactions other than recombination among individual virus strains, specifically via complementation. A defective strain of DENV-1 containing a stop codon in the envelope gene was found to be maintained in both humans and mosquitoes in Myanmar over a period of at least 18 months [137]. Phylogenetic analysis suggested that neither recombination nor stop codon read-through could account for the existence of these strains at such high numbers in multiple hosts. *In vitro* evidence of strain complementation at high MOIs exists for VSV [138], in which no evidence of recombination exists. The relative abundance of low fitness variants of VSV increased with increasing co-infection with high fitness variants, suggesting sharing of viral proteins within a host cell. The potential for cooperative interactions adds layers of complexity to our understanding of how a viral swarm may act in a host and, therefore, how selection acting on a mutant swarm may be fundamentally different from basic population genetics. In addition, the mutant swarm can clearly have suppressive effects on viral fitness as demonstrated by studies with VSV [139] and other RNA viruses such as FMDV [140]. In fact, the whole concept of error catastrophe is based on such suppressive effects [91].

There is limited knowledge about the distribution of fitness values within a given viral swarm at any one given time as a consequence of the dynamic character of a mutant spectrum in nature. The majority of variants within a high fitness population of VSV were found to have fitness values that were on average lower than the population as a whole [124]. This is not surprising given the fact that mutations will generally be deleterious, yet the longevity of these variants in the population is unclear without knowledge of the regularity and nature of cooperative events. Theory tells us that ultimately a phenotypically robust swarm should be selected over a viral swarm with a few highly fit variants surrounded by less fit variants [141–143]. Such a mode of selection is a result of the significance of mutational neighbors in error prone RNA virus replication and has been coined ‘survival of the flattest’ by Wilke *et al.* [144]. Whether or not this concept holds in nature is unclear, since the actual flux of intrahost arboviral populations makes assessing equilibrium generally impossible; yet the existence of widespread complementation and interactive fitness supports a revisiting of such theoretical concepts.

### 3.3. Bottlenecks

Defining the role of bottlenecks in shaping the arbovirus mutant spectrum is crucial to understanding arbovirus evolution. Both theoretical and experimental studies demonstrate that RNA viruses are particularly vulnerable to significant fitness losses from frequent and tight bottlenecks (Muller’s ratchet) due to their inherent propensity to produce deleterious variants [62,145,146]. As a result, frequent bottlenecks should further enhance the evolution of phenotypic robustness. The need for arboviral cycling results in frequent transmission bottlenecks and both transmission size and mode have been shown to have profound effects on mutant swarm evolution [147]. Beyond this, arboviruses may be subject to bottlenecks within hosts and during both emergence in naïve environments and reemergence following seasonal interruptions in transmission. The size and selectivity of these bottlenecks is not well defined and is likely highly variable among both host and viral species.

For arboviruses that utilize mosquito vectors, bottlenecks will occur upon infection of midgut cells, egress from the midgut, infection of parenteral tissues including the salivary glands, and subsequent egress into the salivary secretion during transmission to vertebrate hosts [148–151]. Within vertebrate hosts, bottlenecks similarly occur with the initial establishment of infection, and the subsequent spread through various tissues, particularly the blood for transmission back to the vector. Although bottlenecks within the mosquito are well documented [152,153], the specifics of how they reshape intrahost virus populations are yet to be defined. In a previous study with WNV in *Cx. pipiens*, accumulation of genetic diversity was noted during passage by inoculation when whole bodies were analyzed [71], yet when similar passage was completed using only transmitted virus in the salivary secretion, WNV remained highly genetically homogeneous throughout passage [72]. By bypassing both midgut infection and egress, these studies suggest significant purging of diversity likely occurs during salivary gland infection and/or transmission. Although it has been shown with WNV that mosquitoes can transmit up to 10^6^ plaque forming units of virus [154], it remains unclear what the composition and complexity of the transmitted viral swarm is. Within-host bottlenecks will likely be significantly variable, not just with arboviruses that utilize different vectors, but also among different species and subspecies of the same vector which often demonstrate different levels of vector competence.

Potentially the most significant of all the bottlenecks to which arboviruses are subject are those imposed on viruses which require mechanisms to survive seasonal interruptions in transmission cycles. Phylogenetic studies indicate that most arboviruses are maintained locally, yet the mechanisms for this seasonal maintenance are variable. Some insect vectors may remain persistently infected through winter or other breaks in transmission. For example, ticks infected with Langat virus are still capable of transmitting virus after more than three years [4,155]. Swallow bugs, which are vectors of the alpahvirus *Buggy Creek virus,* can survive for long periods without a vertebrate host and have been found to have a high frequency of infection during winters in the Great Plains in the United States [155]. Many mosquito-borne viruses, including WNV and SLEV, have been shown to be capable of surviving winters in diapausing females [157–160] which were likely initially infected via vertical transmission (VT; [160]), yet rates of VT for these viruses are low (<1.0%; [162,163]). In contrast, rates of VT for bunyaviruses are often relatively high [164,165]. Some populations of *Aedes triseriatus* mosquitoes are capable of transmitting LACV to over 80% of their progeny and venereal transmission also occurs [166]. Mechanisms of overwintering vary not just among viruses but also among species. For example, RRV overwinters in the adults of *Cx. annulirstris* but in the eggs of *Ae. vigilax* [167,168], differences which are likely crucial to the shaping of these viral populations. In addition, many arboviruses also have been shown to form persistent infections in verebrates [169], yet the likelihood of maintaining viremia levels high enough to reinitiate transmission is extremely low.

Ultimately, a virus’ potential to survive and persist following naturally occurring genetic bottlenecks is important to its potential for host range shifts and expansion, and likely has major implications for predicting how viruses will evolve in terms of human susceptibility and pathogenesis. For example, the North American and South American strains of the alphaviruses EEEV differ greatly in their ability to cause neuroinvasive disease in humans [170]. These differences may be partially attributed to how viral swarms have faced differing selective pressures both within disparate hosts and between hosts by differing mechanisms of transmission and maintenance. Without a significant seasonal disruption in transmission for the South American strains, as seen in many places in the U.S. where the North American strains circulate, these populations are clearly subject to different seasonal bottlenecks. In addition, South American EEEV utilizes a broader range of vector species, many of which have more catholic feeding habits than North American vectors [171]; and South American strains utilize primarily ground dwelling animals as amplifying hosts [172]. Similarly, although SLEV has been noted to be distributed by migratory birds, differences in genetic diversity in South American and North American SLEV strains also may be attributed to differences in the role of mammals in South American subpopulations [173]. It remains unclear how these variable selective pressures might ultimately affect human pathogenesis, yet a more detailed understanding of how EEEV populations were differentially shaped could provide insight into the future of SLEV and other arbovirus that persist in ecologically distinct habitats.

## 4. Concluding Remarks

Arboviruses are bound by their need to both infect and cycle between vertebrate and arthropod hosts. It is because of this need that all arboviruses are required to either be generalists or possess some means of phenotypic plasticity. Despite these shared requirements, attempting to generalize findings on arbovirus evolution fails to recognize the enormous diversity in viral genomes and their replication strategies, hosts, and ecology that exists among these viruses.

In conjunction with the outlined need for a more accurate definition of the role of both minority variants and the arboviral swarm in general, a more complete understanding of how these laboratory defined mechanisms translate to functional and, therefore, evolutionary consequences in natural systems is needed. While *in vitro* systems have been highly informative in studying basic concepts, the natural hosts ultimately are required to understand mechanisms of viral adaptation and evolution. Although such *in vivo* experimental studies are beginning to be undertaken, significant expansion of such studies with a focus on host- and virus-specific differences will help to elucidate the unique interactions that shape the evolution of these complex systems.

Phylogenetic studies to date rely exclusively on compilations of consensus sequences from multiple virus isolates. Such studies are highly informative; however, there is a need for large scale evaluation of intrahost genetic diversity both spatially and temporally in nature in order to fully understand the complexity of evolutionary history, the influence of seasonal and within host bottlenecks, and the potential for both phenotypic change and host expansions.
